# A two-stage approach for improved prediction of residue contact maps

**DOI:** 10.1186/1471-2105-7-180

**Published:** 2006-03-30

**Authors:** Alessandro Vullo, Ian Walsh, Gianluca Pollastri

**Affiliations:** 1 School of Computer Science and Informatics, University College Dublin, Belfield, Dublin 4, Ireland

## Abstract

**Background:**

Protein topology representations such as residue contact maps are an important intermediate step towards *ab initio *prediction of protein structure. Although improvements have occurred over the last years, the problem of accurately predicting residue contact maps from primary sequences is still largely unsolved. Among the reasons for this are the unbalanced nature of the problem (with far fewer examples of contacts than non-contacts), the formidable challenge of capturing long-range interactions in the maps, the intrinsic difficulty of mapping one-dimensional input sequences into two-dimensional output maps.

In order to alleviate these problems and achieve improved contact map predictions, in this paper we split the task into two stages: the prediction of a map's principal eigenvector (PE) from the primary sequence; the reconstruction of the contact map from the PE and primary sequence. Predicting the PE from the primary sequence consists in mapping a vector into a vector. This task is less complex than mapping vectors directly into two-dimensional matrices since the size of the problem is drastically reduced and so is the scale length of interactions that need to be learned.

**Results:**

We develop architectures composed of ensembles of two-layered bidirectional recurrent neural networks to classify the components of the PE in 2, 3 and 4 classes from protein primary sequence, predicted secondary structure, and hydrophobicity interaction scales. Our predictor, tested on a non redundant set of 2171 proteins, achieves classification performances of up to 72.6%, 16% above a base-line statistical predictor.

We design a system for the prediction of contact maps from the predicted PE. Our results show that predicting maps through the PE yields sizeable gains especially for long-range contacts which are particularly critical for accurate protein 3D reconstruction. The final predictor's accuracy on a non-redundant set of 327 targets is 35.4% and 19.8% for minimum contact separations of 12 and 24, respectively, when the top length/5 contacts are selected. On the 11 CASP6 Novel Fold targets we achieve similar accuracies (36.5% and 19.7%). This favourably compares with the best automated predictors at CASP6.

**Conclusion:**

Our final system for contact map prediction achieves state-of-the-art performances, and may provide valuable constraints for improved *ab initio *prediction of protein structures. A suite of predictors of structural features, including the PE, and PE-based contact maps, is available at .

## Background

De novo prediction of protein three-dimensional (3D) structure from the primary sequence remains a fundamental and extraordinarily challenging problem [1]. Contact maps, or similar distance restraints have been proposed as intermediate steps between the primary sequence and the 3D structure (e.g. in [2-4]), for various reasons: unlike 3D coordinates, they are invariant to rotations and translations, hence less challenging to predict by machine learning systems [4]; quick, effective algorithms exist to derive 3D structures from them, for instance stochastic optimisation methods [5,6], distance geometry [7,8], or algorithms derived from the NMR literature and elsewhere [9-11]. Numerous methods have been developed for protein residue contact map prediction [2-4,12] and coarse (secondary structure element level) contact map prediction [13], and some improvements are slowly occurring (e.g. in [12], as shown by the CASP6 experiment [14]).

Still, accurate prediction of residue contact maps is far from being achieved and limitations of existing prediction methods have again emerged at CASP6 and from automatic evaluation of structure prediction servers such as EVA [15]. There are various reasons for this: the number of positive and negative examples (contacts vs. non contacts) is strongly unbalanced; the number of examples grows with the squared length of the protein making this a tough computational challenge; capturing long ranged interactions in the primary sequence is difficult, hence grasping an adequate global picture of the map is a formidable problem. For this reason simpler, alternative representations of protein topologies are particularly appealing, provided that they are informative and, especially, predictable (e.g. see [16]).

In this paper we focus on one such representation: the principal eigenvector (PE) of residue contact maps. The PE is a sequence of the same length as a protein's primary sequence. A vast machinery of tools for sequence processing is available (see e.g. [17] for a review). Moreover, recently [18] a branch-and-bound algorithm was described that is capable of reconstructing the contact map from the exact PE, at least for single domain proteins of up to 120 amino acids. This means that the PE contains most of the information encoded in the contact map. Predicting the PE is thus interesting: it leads to a drastic reduction in the size of the problem compared to two-dimensional contact maps, i.e. considerable data compression, and also a reduction in the scale length of interactions that need to be learned; contact maps may be derived from the PE by modifying the reconstruction algorithm in [18] to deal with noise in the PE; alternatively the PE may be adopted as an additional input feature to systems for the direct prediction of contact maps (such as [4]); information contained in the PE may be used, in combination with other constraints, to guide the search for optimal 3D configurations; predicted PE may prove useful to identify domains, as in [19], and discussed in [20].

In this paper, we model the problem of inferring the PE as a classification task with multiple classes. We use machine learning methods to map amino acids into their corresponding component of the principal eigenvector. Similarly to [21], we adopt bidirectional recurrent neural networks (BRNNs) [22] with shortcut connections, accurate coding of input profiles obtained from multiple sequence alignments, secondary structure predictions, second stage filtering by recurrent neural networks, and finally large-scale ensembles of predictors. Our models classify correctly up to 72.6% residues, 16% above a base-line statistical predictor always assigning a residue to the most numerous PE class.

To prove that these levels can lead to improved contact maps, we incorporate the predicted PE into a state-of-the-art system for contact map prediction [4,13]. Our tests show that the PE yields sizeable gains, and that these gains are especially significant for long-ranged contacts, which are known to be both harder to predict and critical for accurate 3D reconstruction.

## Results and discussion

### Principal eigenvector prediction

We evaluate model performances using different prediction indices. If the task is the prediction of the eigenvector components λ¯x¯
 MathType@MTEF@5@5@+=feaafiart1ev1aaatCvAUfKttLearuWrP9MDH5MBPbIqV92AaeXatLxBI9gBaebbnrfifHhDYfgasaacH8akY=wiFfYdH8Gipec8Eeeu0xXdbba9frFj0=OqFfea0dXdd9vqai=hGuQ8kuc9pgc9s8qqaq=dirpe0xb9q8qiLsFr0=vr0=vr0dc8meaabaqaciaacaGaaeqabaqabeGadaaakeaacuaH7oaBgaqeaiqbdIha4zaaraaaaa@3009@_*i *_(see the methods section for definitions) in *m *classes, we measure: *Q*_*m*_, or overall percentage of correctly predicted amino acids; the set *Q*_0_, ..., *Q*_*m*-1_, where each *Q*_*j *_is the percentage of correctly classified amino acids whose eigenvector component belongs to interval *I*_*j*+1_; an analogous of the SOV measure [23] adapted for the case of *m *classes. The intent in this case is to measure the quality of prediction over contiguous segments of amino acids belonging to the same class. Finally, we compare our methodology with a base-line predictor that assigns each amino acid to its most frequently occurring class (as for instance in [24-26]).

We train different ensembles of BRNNs. Differences depend on whether or not we use output filtering by second stage networks (Eq.6) and whether or not the input encoding includes predicted secondary structure from Porter [21] and the hydrophobicity interaction scale in [20] (Table I, column 2). Tables 1, 2 and 3 show respectively estimated performance indices for classification in two, three and four classes. The first three columns indicate whether secondary structure, hydrophobicity profile and second stage filtering are employed in the network ensemble (see table legends for details).

In all multi-class prediction cases, the best network ensemble shows an increment of global predictive accuracy of ≈ 16% with respect to the base-line predictor. The SOV and the overall accuracy increase using filters and augmenting the number of input features with hydrophobicity scales (marginally) and secondary structure (significantly). Interestingly, predicted secondary structure is a valuable feature: in all cases, using true secondary structures results in only moderate improvements with respect to the performance obtained with secondary structure predicted by Porter [21] (data not shown).

In the 2-class problem, *Q*_2 _exceeds 72% with the two classes almost equally well predicted. In this case, the network with full features finds a nearly optimal (Bayesian) decision threshold. This is not surprising because the threshold on the eigenvector component was chosen so as to divide the training set values in two equally distributed halves. The 3- and 4-class prediction problems are more difficult to solve, but the observed improvements over the baseline predictor are roughly the same as in the 2-class case. Strong improvements over the base-line predictor are observed especially for the intermediate classes (*Q*_1 _in Table 2, *Q*_1 _and *Q*_2 _in Table 3). Interestingly, these classes are more difficult to predict even if all classes are nearly equally distributed. This is possibly because boundary classes (*Q*_0 _and *Q*_*m*-1_) correspond to well-defined situations, i.e. isolated residues or residues with high connectivity, for which clear signal exists in the data. A typical example of 4-class PE prediction is shown in figure 2.

### Contact map prediction from predicted PE

As a final step, we test the possibility of directly using the information encoded in the PE to improve state-of-the-art residue contact map predictors. We choose the model based on DAG-RNNs described in [4] and [13]. This model was among the most successful contact map predictors at the CASP5 competition [27]. The architecture we adopt is identical to the one described in [13] and used at CASP5, except for the presence of shortcut connections and for the ensembling technique (see methods section). These differences allow a substantially (roughly 5-fold) faster training, and yield marginally improved results compared to [13] when the same input features and same training/test sets are adopted (not shown).

To ensure fairness, here we retrain DAG-RNNs from scratch using the same training and testing sets used to predict the PE. The sets are first processed to remove sequences longer than 200 amino acids (for computational reasons, as in [13]), leaving 1275 proteins in the training set and 327 proteins in the test set. Two amino acids are defined as being in contact if the distance between their *C*_*α *_is below a contact threshold. We consider two different contact thresholds: 8 and 12 Å. For comparison purposes, we encode each pair (*i*, *j*) of amino acids in the input by four different features: a 20 × 20 matrix representing the probability distribution of pairs of amino acids observed in the two corresponding columns of the alignment (MA); MA plus the actual discretised 4-class PE component for both residue *i *and *j *(MA_PE); MA plus the actual secondary structure (3 classes) and binary thresholded (at 25%) relative solvent accessibility (MA_SS_ACC); and finally, the previous feature plus the actual 4-class PE components (MA_SS_ACC_PE). We train 8 predictors, with the same architecture, one for each input feature and contact threshold.

Differently from the training phase, testing takes place by encoding each pair (*i*, *j*) on input with the *predicted *4-class PE component as given by the filtered ensemble of BRNNs using profiles, predicted secondary structure and hydrophobicity profiles (table 3, row P-H-F). Secondary structure and solvent accessibility information input into the DAG-RNN is also predicted during testing. These predictions are obtained from an architecture identical to the one adopted to predict the PE, and trained on the same training set. Hence the protocol we adopt leads to fully realistic results, since no protein in the the test set shows significant sequence similarity to any of the structures used to train the contact map predictor and all the underlying feature predictors.

Tables 4, 5, 6 and 7 show performance indices for all the 8 networks. Indices considered are: accuracy *P *= *TP*/(*TP *+ *FP*), with *TP *= true positives and *FP *= false positives; coverage *R *= *TP*/(*TP *+ *FN*), with *FN *= false negatives; *F*_1_, defined as the harmonic mean of accuracy and coverage (*F*_1 _= 2*PR*/(*P *+ *R*)); *P*_*nc*_, the percentage of correctly predicted non-contacts. Performances are computed for three different sets of contacts, based on the separation of two residues in the linear sequence: |*i *- *j*| ≥ {6,12, 24}. In tables 4 and 6 we report *P*, *R *and *F*_1 _when the threshold between contacts and non-contacts is set to 0.5. In tables 5 and 7, for consistency with CASP assessment rules [14], we report *P *and *R *when only the top *N*/5 and *N*/2 contacts are considered, *N *being the length of the protein. In this case contacts are ranked, and top contacts are selected, based on their expected probability as estimated by the predictor.

As evident from the tables, the introduction of PE predictions increases the *F*_1 _measure in all cases. This is true for both 8 and 12 Å maps, and for all separation thresholds. An improvent is observed both in the MA_PE vs. MA case and in the MA_SS_ACC_PE vs. MA_SS_ACC case. In all cases the introduction of the predicted PE yields larger performance gains than secondary structure and solvent accessibility combined. Interestingly, the gains become more significant for longer range contacts. For instance for |*i *- *j*| ≥ 24 *F*_1 _grows from 0.3% to 4.1% at 8Å and from 5.3% to 16.8% at 12Å (MA_SS_ACC_PE vs. MA_SS_ACC). PE-based networks are more confident away from the main diagonal (a typical example is shown in figure 3), with a better balance between false positives and false negatives.

When we take into account only small numbers (*N*/5 and *N*/2) of contacts considered most likely by the predictor, the gains become less marked, but remain significant, especially for longer range contacts: for |*i *- *j*| ≥ 24 at 8Å, when considering the top *N*/5 contacts, *P *grows from 14.1% to 19.6% in the MA vs. MA_PE case and from 17.8% to 19.8% in the in the MA_SS_ACC vs. MA_SS_ACC_PE case. Similar gains (from 43.3% to 47.9% and from 43.7% to 49.9%, respectively) are observed for the 12Å predictors.

Residue contact map predictors at CASP6 [14] were evaluated on a small set (11) of Novel Fold targets. The performances of the best system (group RR301) on the top *N*/5 contacts were 24% and 22% (accuracy) and 5.6% and 5% (coverage) for minimum residue separations of 12 and 24, respectively. Although the statistical relevance of a set of only 11 targets is limited, our predictor's accuracy on it compares favourably with the best CASP6 predictors, achieving 36.5% accuracy and 9.8% coverage for separation of at least 12 and 19.6% accuracy and 5.4% coverage for separation of at least 24.

## Conclusion

We developed sophisticated predictors of a novel sequential feature of protein structure: the principal eigenvector of residue contact maps. Our predictors classify correctly up to 72.6% of residues, and show large gains over simple base-line statistical predictors.

We showed that predicted principal eigenvectors can be effectively used as an additional input feature to a state-of-the-art method for contact map prediction, yielding sizeable gains especially for long-range contacts which are particularly critical for accurate protein 3D reconstruction.

These results suggest a number of futher points to investigate:

• The algorithm in [18] may be directly tested in noisy contexts, and extended to increase its robustness. This may give rise to an alternative pipeline for the prediction of contact maps.

• The PE could be used directly to improve protein domain predictors [19,20].

• PE-based maps may be adopted to guide the *ab initio *reconstruction of quick, draft *C*_*α *_traces, for instance using a stochastic search algorithm similar to [5].

• Residue coordination number correlates well with the PE – as such, predicted coordination number [26] may yield similar gains to contact map prediction, while providing a more intuitive structural representation of a protein.

Ultimately, the third point is the most crucial test of the validity of our approach. Even if the 3D models produced were fairly coarse, they might be provide a valuable source of information, for instance to identify protein functions more accurately than it would be possible by sequence alone [28]. Although training a contact map prediction system is computationally expensive, once training is over, generating predictions is fast. Even on a small cluster of machines, this may allow multi-genomic scale structural prediction efforts in manageable times.

## Methods

The contact map of a protein with *N *amino acids is a symmetric *N *× *N *matrix *C*, with elements *C*_*ij *_defined as:

Cij={1if amino acid i and j are in contact0otherwise     (1)
 MathType@MTEF@5@5@+=feaafiart1ev1aaatCvAUfKttLearuWrP9MDH5MBPbIqV92AaeXatLxBI9gBaebbnrfifHhDYfgasaacH8akY=wiFfYdH8Gipec8Eeeu0xXdbba9frFj0=OqFfea0dXdd9vqai=hGuQ8kuc9pgc9s8qqaq=dirpe0xb9q8qiLsFr0=vr0=vr0dc8meaabaqaciaacaGaaeqabaqabeGadaaakeaacqWGdbWqdaWgaaWcbaGaemyAaKMaemOAaOgabeaakiabg2da9maaceaabaqbaeaabiGaaaqaaiabigdaXaqaaiabbMgaPjabbAgaMjabbccaGiabbggaHjabb2gaTjabbMgaPjabb6gaUjabb+gaVjabbccaGiabbggaHjabbogaJjabbMgaPjabbsgaKjabbccaGiabbMgaPjabbccaGiabbggaHjabb6gaUjabbsgaKjabbccaGiabbQgaQjabbccaGiabbggaHjabbkhaYjabbwgaLjabbccaGiabbMgaPjabb6gaUjabbccaGiabbogaJjabb+gaVjabb6gaUjabbsha0jabbggaHjabbogaJjabbsha0bqaaiabicdaWaqaaiabb+gaVjabbsha0jabbIgaOjabbwgaLjabbkhaYjabbEha3jabbMgaPjabbohaZjabbwgaLbaacaWLjaGaaCzcamaabmaabaGaeGymaedacaGLOaGaayzkaaaacaGL7baaaaa@70CC@

We define two amino acids as being in contact if the distance between their *C*_*α *_is less than a given threshold. For the definition of the PE we adopt a fixed 8 Å threshold, while in the contact map prediction stage we test 8 Å and 12 Å thresholds. Alternative definitions are possible, for instance based on different mutual *C*_*α *_distances (normally in the 7–12 Å range), or on *C*_*β*_-*C*_*β *_atom distances (normally 6.5–8 Å), or on the minimal distance between two atoms belonging to the side-chain or backbone of the two residues (commonly 4.5 Å).

Let λ(*C*) = {λ : *Cx *= λ*x*} be the spectrum of *C*, S
 MathType@MTEF@5@5@+=feaafiart1ev1aaatCvAUfKttLearuWrP9MDH5MBPbIqV92AaeXatLxBI9gBamrtHrhAL1wy0L2yHvtyaeHbnfgDOvwBHrxAJfwnaebbnrfifHhDYfgasaacH8akY=wiFfYdH8Gipec8Eeeu0xXdbba9frFj0=OqFfea0dXdd9vqai=hGuQ8kuc9pgc9s8qqaq=dirpe0xb9q8qiLsFr0=vr0=vr0dc8meaabaqaciaacaGaaeqabaWaaeGaeaaakeaaimaacqWFse=uaaa@3845@_λ _= {*x *: *Cx *= λ*x*} the corresponding eigenspace and λ¯
 MathType@MTEF@5@5@+=feaafiart1ev1aaatCvAUfKttLearuWrP9MDH5MBPbIqV92AaeXatLxBI9gBaebbnrfifHhDYfgasaacH8akY=wiFfYdH8Gipec8Eeeu0xXdbba9frFj0=OqFfea0dXdd9vqai=hGuQ8kuc9pgc9s8qqaq=dirpe0xb9q8qiLsFr0=vr0=vr0dc8meaabaqaciaacaGaaeqabaqabeGadaaakeaacuaH7oaBgaqeaaaa@2E78@ = max{λ ∈ λ(*C*)} the largest eigenvalue of *C*. The principal eigenvector of *C*, x¯
 MathType@MTEF@5@5@+=feaafiart1ev1aaatCvAUfKttLearuWrP9MDH5MBPbIqV92AaeXatLxBI9gBaebbnrfifHhDYfgasaacH8akY=wiFfYdH8Gipec8Eeeu0xXdbba9frFj0=OqFfea0dXdd9vqai=hGuQ8kuc9pgc9s8qqaq=dirpe0xb9q8qiLsFr0=vr0=vr0dc8meaabaqaciaacaGaaeqabaqabeGadaaakeaacuWG4baEgaqeaaaa@2E3D@, is the eigenvector corresponding to λ¯
 MathType@MTEF@5@5@+=feaafiart1ev1aaatCvAUfKttLearuWrP9MDH5MBPbIqV92AaeXatLxBI9gBaebbnrfifHhDYfgasaacH8akY=wiFfYdH8Gipec8Eeeu0xXdbba9frFj0=OqFfea0dXdd9vqai=hGuQ8kuc9pgc9s8qqaq=dirpe0xb9q8qiLsFr0=vr0=vr0dc8meaabaqaciaacaGaaeqabaqabeGadaaakeaacuaH7oaBgaqeaaaa@2E78@. x¯
 MathType@MTEF@5@5@+=feaafiart1ev1aaatCvAUfKttLearuWrP9MDH5MBPbIqV92AaeXatLxBI9gBaebbnrfifHhDYfgasaacH8akY=wiFfYdH8Gipec8Eeeu0xXdbba9frFj0=OqFfea0dXdd9vqai=hGuQ8kuc9pgc9s8qqaq=dirpe0xb9q8qiLsFr0=vr0=vr0dc8meaabaqaciaacaGaaeqabaqabeGadaaakeaacuWG4baEgaqeaaaa@2E3D@ can also be expressed as the argument which maximises the Rayleigh quotient:

∀x∈Sλ:xTCxxTx≤x¯TCx¯x¯Tx¯     (2)
 MathType@MTEF@5@5@+=feaafiart1ev1aaatCvAUfKttLearuWrP9MDH5MBPbIqV92AaeXatLxBI9gBamrtHrhAL1wy0L2yHvtyaeHbnfgDOvwBHrxAJfwnaebbnrfifHhDYfgasaacH8akY=wiFfYdH8Gipec8Eeeu0xXdbba9frFj0=OqFfea0dXdd9vqai=hGuQ8kuc9pgc9s8qqaq=dirpe0xb9q8qiLsFr0=vr0=vr0dc8meaabaqaciaacaGaaeqabaWaaeGaeaaakeaacqGHaiIicqWG4baEcqGHiiIZimaacqWFse=udaWgaaWcbaGaeq4UdWgabeaakiabcQda6maalaaabaGaemiEaG3aaWbaaSqabeaacqWGubavaaGccqWGdbWqcqWG4baEaeaacqWG4baEdaahaaWcbeqaaiabdsfaubaakiabdIha4baacqGHKjYOdaWcaaqaaiqbdIha4zaaraWaaWbaaSqabeaacqWGubavaaGccqWGdbWqcuWG4baEgaqeaaqaaiqbdIha4zaaraWaaWbaaSqabeaacqWGubavaaGccuWG4baEgaqeaaaacaWLjaGaaCzcamaabmaabaGaeGOmaidacaGLOaGaayzkaaaaaa@5872@

Eigenvectors are usually normalised by requiring their norm to be 1, e.g. ||*x*||_2 _= 1 ∀*x *∈ S
 MathType@MTEF@5@5@+=feaafiart1ev1aaatCvAUfKttLearuWrP9MDH5MBPbIqV92AaeXatLxBI9gBamrtHrhAL1wy0L2yHvtyaeHbnfgDOvwBHrxAJfwnaebbnrfifHhDYfgasaacH8akY=wiFfYdH8Gipec8Eeeu0xXdbba9frFj0=OqFfea0dXdd9vqai=hGuQ8kuc9pgc9s8qqaq=dirpe0xb9q8qiLsFr0=vr0=vr0dc8meaabaqaciaacaGaaeqabaWaaeGaeaaakeaaimaacqWFse=uaaa@3845@_λ_. Since *C *is an adjacency (real, symmetric) matrix, its eigenvalues are real. Since it is a normal matrix (*A*^*H*^*A *= *AA*^*H*^), its eigenvectors are orthogonal. Other basic properties can also be proven: the principal eigenvalue is positive; non-zero components of x¯
 MathType@MTEF@5@5@+=feaafiart1ev1aaatCvAUfKttLearuWrP9MDH5MBPbIqV92AaeXatLxBI9gBaebbnrfifHhDYfgasaacH8akY=wiFfYdH8Gipec8Eeeu0xXdbba9frFj0=OqFfea0dXdd9vqai=hGuQ8kuc9pgc9s8qqaq=dirpe0xb9q8qiLsFr0=vr0=vr0dc8meaabaqaciaacaGaaeqabaqabeGadaaakeaacuWG4baEgaqeaaaa@2E3D@ have all the same sign [29]. Without loss of generality, we can assume they are positive, as in [18].

Ideally, prediction of the PE should be formulated as a sequential regression task in which each amino acid is mapped into its corresponding component of the PE. Here we consider two variations to the original problem. First, we model it as a classification task with multiple classes. Second, we predict the magnified eigenvector, i.e. λ¯x¯
 MathType@MTEF@5@5@+=feaafiart1ev1aaatCvAUfKttLearuWrP9MDH5MBPbIqV92AaeXatLxBI9gBaebbnrfifHhDYfgasaacH8akY=wiFfYdH8Gipec8Eeeu0xXdbba9frFj0=OqFfea0dXdd9vqai=hGuQ8kuc9pgc9s8qqaq=dirpe0xb9q8qiLsFr0=vr0=vr0dc8meaabaqaciaacaGaaeqabaqabeGadaaakeaacuaH7oaBgaqeaiqbdIha4zaaraaaaa@3009@ instead of x¯
 MathType@MTEF@5@5@+=feaafiart1ev1aaatCvAUfKttLearuWrP9MDH5MBPbIqV92AaeXatLxBI9gBaebbnrfifHhDYfgasaacH8akY=wiFfYdH8Gipec8Eeeu0xXdbba9frFj0=OqFfea0dXdd9vqai=hGuQ8kuc9pgc9s8qqaq=dirpe0xb9q8qiLsFr0=vr0=vr0dc8meaabaqaciaacaGaaeqabaqabeGadaaakeaacuWG4baEgaqeaaaa@2E3D@. Modelling regression problems as multi-class classifications is common practice, for instance in closely related tasks such as the prediction of protein solvent accessibility [26,30,31]. Predicting magnified eigenvector components has some advantages: by doing so we are simultaneously estimating the eigenvector components and the corresponding eigenvalue (as the norm of x¯
 MathType@MTEF@5@5@+=feaafiart1ev1aaatCvAUfKttLearuWrP9MDH5MBPbIqV92AaeXatLxBI9gBaebbnrfifHhDYfgasaacH8akY=wiFfYdH8Gipec8Eeeu0xXdbba9frFj0=OqFfea0dXdd9vqai=hGuQ8kuc9pgc9s8qqaq=dirpe0xb9q8qiLsFr0=vr0=vr0dc8meaabaqaciaacaGaaeqabaqabeGadaaakeaacuWG4baEgaqeaaaa@2E3D@ is equal to 1); the main eigenvalue correlates well with the protein length (correlation of 0.62 on our training set), hence it is likely predictable. An estimate of the eigenvalue will in general be needed when attempting to predict contact maps from the PE, either by using an algorithm similar to the one in [18], or more in general by attempting to satisfy the constraint:

|∑jCijx¯j−λ¯x¯i|=0     (3)
 MathType@MTEF@5@5@+=feaafiart1ev1aaatCvAUfKttLearuWrP9MDH5MBPbIqV92AaeXatLxBI9gBaebbnrfifHhDYfgasaacH8akY=wiFfYdH8Gipec8Eeeu0xXdbba9frFj0=OqFfea0dXdd9vqai=hGuQ8kuc9pgc9s8qqaq=dirpe0xb9q8qiLsFr0=vr0=vr0dc8meaabaqaciaacaGaaeqabaqabeGadaaakeaacqGG8baFdaaeqbqaaiabdoeadnaaBaaaleaacqWGPbqAcqWGQbGAaeqaaOGafmiEaGNbaebadaWgaaWcbaGaemOAaOgabeaakiabgkHiTiqbeU7aSzaaraGafmiEaGNbaebadaWgaaWcbaGaemyAaKgabeaakiabcYha8jabg2da9iabicdaWaWcbaGaemOAaOgabeqdcqGHris5aOGaaCzcaiaaxMaadaqadaqaaiabiodaZaGaayjkaiaawMcaaaaa@45E7@

Formally, the PE prediction task consists in learning a mapping *f*(·) : ℐ
 MathType@MTEF@5@5@+=feaafiart1ev1aaatCvAUfKttLearuWrP9MDH5MBPbIqV92AaeXatLxBI9gBamrtHrhAL1wy0L2yHvtyaeHbnfgDOvwBHrxAJfwnaebbnrfifHhDYfgasaacH8akY=wiFfYdH8Gipec8Eeeu0xXdbba9frFj0=OqFfea0dXdd9vqai=hGuQ8kuc9pgc9s8qqaq=dirpe0xb9q8qiLsFr0=vr0=vr0dc8meaabaqaciaacaGaaeqabaWaaeGaeaaakeaaimaacqWFqessaaa@3769@ → O
 MathType@MTEF@5@5@+=feaafiart1ev1aaatCvAUfKttLearuWrP9MDH5MBPbIqV92AaeXatLxBI9gBamrtHrhAL1wy0L2yHvtyaeHbnfgDOvwBHrxAJfwnaebbnrfifHhDYfgasaacH8akY=wiFfYdH8Gipec8Eeeu0xXdbba9frFj0=OqFfea0dXdd9vqai=hGuQ8kuc9pgc9s8qqaq=dirpe0xb9q8qiLsFr0=vr0=vr0dc8meaabaqaciaacaGaaeqabaWaaeGaeaaakeaaimaacqWFoe=taaa@383D@ from the space ℐ
 MathType@MTEF@5@5@+=feaafiart1ev1aaatCvAUfKttLearuWrP9MDH5MBPbIqV92AaeXatLxBI9gBamrtHrhAL1wy0L2yHvtyaeHbnfgDOvwBHrxAJfwnaebbnrfifHhDYfgasaacH8akY=wiFfYdH8Gipec8Eeeu0xXdbba9frFj0=OqFfea0dXdd9vqai=hGuQ8kuc9pgc9s8qqaq=dirpe0xb9q8qiLsFr0=vr0=vr0dc8meaabaqaciaacaGaaeqabaWaaeGaeaaakeaaimaacqWFqessaaa@3769@ of labelled input sequences to the space O
 MathType@MTEF@5@5@+=feaafiart1ev1aaatCvAUfKttLearuWrP9MDH5MBPbIqV92AaeXatLxBI9gBamrtHrhAL1wy0L2yHvtyaeHbnfgDOvwBHrxAJfwnaebbnrfifHhDYfgasaacH8akY=wiFfYdH8Gipec8Eeeu0xXdbba9frFj0=OqFfea0dXdd9vqai=hGuQ8kuc9pgc9s8qqaq=dirpe0xb9q8qiLsFr0=vr0=vr0dc8meaabaqaciaacaGaaeqabaWaaeGaeaaakeaaimaacqWFoe=taaa@383D@ of labelled output sequences. In practice, we want to predict a sequence of labels *O *= (*o*_1_, ..., *o*_*N*_), for a given sequence of inputs *I *= (*i*_1_, ..., *i*_*N*_), where each *i*_*j *_∈ *I *is the input coding of the amino acid in position *j*. For PE prediction, we assume that there is a range *R *including all magnified eigenvector components, i.e. ∀*j*, λ¯x¯
 MathType@MTEF@5@5@+=feaafiart1ev1aaatCvAUfKttLearuWrP9MDH5MBPbIqV92AaeXatLxBI9gBaebbnrfifHhDYfgasaacH8akY=wiFfYdH8Gipec8Eeeu0xXdbba9frFj0=OqFfea0dXdd9vqai=hGuQ8kuc9pgc9s8qqaq=dirpe0xb9q8qiLsFr0=vr0=vr0dc8meaabaqaciaacaGaaeqabaqabeGadaaakeaacuaH7oaBgaqeaiqbdIha4zaaraaaaa@3009@_*j *_∈ *R*, and we divide the range *R *into a series of *m *disjoint intervals, i.e. R=∪k=1mRk
 MathType@MTEF@5@5@+=feaafiart1ev1aaatCvAUfKttLearuWrP9MDH5MBPbIqV92AaeXatLxBI9gBaebbnrfifHhDYfgasaacH8akY=wiFfYdH8Gipec8Eeeu0xXdbba9frFj0=OqFfea0dXdd9vqai=hGuQ8kuc9pgc9s8qqaq=dirpe0xb9q8qiLsFr0=vr0=vr0dc8meaabaqaciaacaGaaeqabaqabeGadaaakeaacqWGsbGucqGH9aqpdaWeWaqaaiabdkfasnaaBaaaleaacqWGRbWAaeqaaaqaaiabdUgaRjabg2da9iabigdaXaqaaiabd2gaTbqdcqWIQisvaaaa@37D7@. We can represent each output label *o*_*j *_as belonging to an alphabet of *m *symbols, i.e. *o*_*j *_∈ Σ = {1, ..., *m*}, and *o*_*j *_corresponds to the class or interval *R*_*k *_in which the value of the *j*-th magnified eigenvector component falls: *o*_*j *_= *k *⇔ λ¯x¯
 MathType@MTEF@5@5@+=feaafiart1ev1aaatCvAUfKttLearuWrP9MDH5MBPbIqV92AaeXatLxBI9gBaebbnrfifHhDYfgasaacH8akY=wiFfYdH8Gipec8Eeeu0xXdbba9frFj0=OqFfea0dXdd9vqai=hGuQ8kuc9pgc9s8qqaq=dirpe0xb9q8qiLsFr0=vr0=vr0dc8meaabaqaciaacaGaaeqabaqabeGadaaakeaacuaH7oaBgaqeaiqbdIha4zaaraaaaa@3009@_*j *_∈ *R*_*k*_.

### Predictive architecture for the PE

To learn the mapping between our inputs ℐ
 MathType@MTEF@5@5@+=feaafiart1ev1aaatCvAUfKttLearuWrP9MDH5MBPbIqV92AaeXatLxBI9gBamrtHrhAL1wy0L2yHvtyaeHbnfgDOvwBHrxAJfwnaebbnrfifHhDYfgasaacH8akY=wiFfYdH8Gipec8Eeeu0xXdbba9frFj0=OqFfea0dXdd9vqai=hGuQ8kuc9pgc9s8qqaq=dirpe0xb9q8qiLsFr0=vr0=vr0dc8meaabaqaciaacaGaaeqabaWaaeGaeaaakeaaimaacqWFqessaaa@3769@ and outputs O
 MathType@MTEF@5@5@+=feaafiart1ev1aaatCvAUfKttLearuWrP9MDH5MBPbIqV92AaeXatLxBI9gBamrtHrhAL1wy0L2yHvtyaeHbnfgDOvwBHrxAJfwnaebbnrfifHhDYfgasaacH8akY=wiFfYdH8Gipec8Eeeu0xXdbba9frFj0=OqFfea0dXdd9vqai=hGuQ8kuc9pgc9s8qqaq=dirpe0xb9q8qiLsFr0=vr0=vr0dc8meaabaqaciaacaGaaeqabaWaaeGaeaaakeaaimaacqWFoe=taaa@383D@ we use a two-layered architecture composed of Bidirectional Recurrent Neural Networks (BRNN) [22] (also known as 1D-RNN, e.g. in [13]) of the same length as the amino acid sequence. Similarly to [21] we use BRNNs with *shortcut connections*. In these BRNNs, connections along the forward and backward hidden chains span more than 1-residue intervals, creating shorter paths between inputs and outputs. These networks take the form:

oj=N(O)(ij,hj(F),hj(B))hj(F)=N(F)(ij,hj−1(F),…,hj−S(F))hj(B)=N(B)(ij,hj+1(B),…,hj+S(B))j=1,…,N
 MathType@MTEF@5@5@+=feaafiart1ev1aaatCvAUfKttLearuWrP9MDH5MBPbIqV92AaeXatLxBI9gBamrtHrhAL1wy0L2yHvtyaeHbnfgDOvwBHrxAJfwnaebbnrfifHhDYfgasaacH8akY=wiFfYdH8Gipec8Eeeu0xXdbba9frFj0=OqFfea0dXdd9vqai=hGuQ8kuc9pgc9s8qqaq=dirpe0xb9q8qiLsFr0=vr0=vr0dc8meaabaqaciaacaGaaeqabaWaaeGaeaaakeaafaqabeabbaaaaeaacqWGVbWBdaWgaaWcbaGaemOAaOgabeaakiabg2da9GWaaiab=1q8onaaCaaaleqabaWaaeWaaeaacqWGpbWtaiaawIcacaGLPaaaaaGcdaqadaqaaiabdMgaPnaaBaaaleaacqWGQbGAaeqaaOGaeiilaWIaemiAaG2aa0baaSqaaiabdQgaQbqaamaabmaabaGaemOrayeacaGLOaGaayzkaaaaaOGaeiilaWIaemiAaG2aa0baaSqaaiabdQgaQbqaamaabmaabaGaemOqaieacaGLOaGaayzkaaaaaaGccaGLOaGaayzkaaaabaGaemiAaG2aa0baaSqaaiabdQgaQbqaamaabmaabaGaemOrayeacaGLOaGaayzkaaaaaOGaeyypa0Jae8xdX70aaWbaaSqabeaadaqadaqaaiabdAeagbGaayjkaiaawMcaaaaakmaabmaabaGaemyAaK2aaSbaaSqaaiabdQgaQbqabaGccqGGSaalcqWGObaAdaqhaaWcbaGaemOAaOMaeyOeI0IaeGymaedabaWaaeWaaeaacqWGgbGraiaawIcacaGLPaaaaaGccqGGSaalcqWIMaYscqGGSaalcqWGObaAdaqhaaWcbaGaemOAaOMaeyOeI0Iaem4uamfabaWaaeWaaeaacqWGgbGraiaawIcacaGLPaaaaaaakiaawIcacaGLPaaaaeaacqWGObaAdaqhaaWcbaGaemOAaOgabaWaaeWaaeaacqWGcbGqaiaawIcacaGLPaaaaaGccqGH9aqpcqWFneVtdaahaaWcbeqaamaabmaabaGaemOqaieacaGLOaGaayzkaaaaaOWaaeWaaeaacqWGPbqAdaWgaaWcbaGaemOAaOgabeaakiabcYcaSiabdIgaOnaaDaaaleaacqWGQbGAcqGHRaWkcqaIXaqmaeaadaqadaqaaiabdkeacbGaayjkaiaawMcaaaaakiabcYcaSiablAciljabcYcaSiabdIgaOnaaDaaaleaacqWGQbGAcqGHRaWkcqWGtbWuaeaadaqadaqaaiabdkeacbGaayjkaiaawMcaaaaaaOGaayjkaiaawMcaaaqaaiabdQgaQjabg2da9iabigdaXiabcYcaSiablAciljabcYcaSiabd6eaobaaaaa@9C9A@

where hj(F)
 MathType@MTEF@5@5@+=feaafiart1ev1aaatCvAUfKttLearuWrP9MDH5MBPbIqV92AaeXatLxBI9gBaebbnrfifHhDYfgasaacH8akY=wiFfYdH8Gipec8Eeeu0xXdbba9frFj0=OqFfea0dXdd9vqai=hGuQ8kuc9pgc9s8qqaq=dirpe0xb9q8qiLsFr0=vr0=vr0dc8meaabaqaciaacaGaaeqabaqabeGadaaakeaacqWGObaAdaqhaaWcbaGaemOAaOgabaGaeiikaGIaemOrayKaeiykaKcaaaaa@3256@ and hj(B)
 MathType@MTEF@5@5@+=feaafiart1ev1aaatCvAUfKttLearuWrP9MDH5MBPbIqV92AaeXatLxBI9gBaebbnrfifHhDYfgasaacH8akY=wiFfYdH8Gipec8Eeeu0xXdbba9frFj0=OqFfea0dXdd9vqai=hGuQ8kuc9pgc9s8qqaq=dirpe0xb9q8qiLsFr0=vr0=vr0dc8meaabaqaciaacaGaaeqabaqabeGadaaakeaacqWGObaAdaqhaaWcbaGaemOAaOgabaGaeiikaGIaemOqaiKaeiykaKcaaaaa@324E@ are forward and backward chains of hidden vectors with h0(F)=hN+1(B)=0
 MathType@MTEF@5@5@+=feaafiart1ev1aaatCvAUfKttLearuWrP9MDH5MBPbIqV92AaeXatLxBI9gBaebbnrfifHhDYfgasaacH8akY=wiFfYdH8Gipec8Eeeu0xXdbba9frFj0=OqFfea0dXdd9vqai=hGuQ8kuc9pgc9s8qqaq=dirpe0xb9q8qiLsFr0=vr0=vr0dc8meaabaqaciaacaGaaeqabaqabeGadaaakeaacqWGObaAdaqhaaWcbaGaeGimaadabaGaeiikaGIaemOrayKaeiykaKcaaOGaeyypa0JaemiAaG2aa0baaSqaaiabd6eaojabgUcaRiabigdaXaqaaiabcIcaOiabdkeacjabcMcaPaaakiabg2da9iabicdaWaaa@3C31@. We parametrise the output update, forward update and backward update functions (respectively N
 MathType@MTEF@5@5@+=feaafiart1ev1aaatCvAUfKttLearuWrP9MDH5MBPbIqV92AaeXatLxBI9gBamrtHrhAL1wy0L2yHvtyaeHbnfgDOvwBHrxAJfwnaebbnrfifHhDYfgasaacH8akY=wiFfYdH8Gipec8Eeeu0xXdbba9frFj0=OqFfea0dXdd9vqai=hGuQ8kuc9pgc9s8qqaq=dirpe0xb9q8qiLsFr0=vr0=vr0dc8meaabaqaciaacaGaaeqabaWaaeGaeaaakeaaimaacqWFneVtaaa@383B@^(*O*)^, N
 MathType@MTEF@5@5@+=feaafiart1ev1aaatCvAUfKttLearuWrP9MDH5MBPbIqV92AaeXatLxBI9gBamrtHrhAL1wy0L2yHvtyaeHbnfgDOvwBHrxAJfwnaebbnrfifHhDYfgasaacH8akY=wiFfYdH8Gipec8Eeeu0xXdbba9frFj0=OqFfea0dXdd9vqai=hGuQ8kuc9pgc9s8qqaq=dirpe0xb9q8qiLsFr0=vr0=vr0dc8meaabaqaciaacaGaaeqabaWaaeGaeaaakeaaimaacqWFneVtaaa@383B@^(*F*) ^and N
 MathType@MTEF@5@5@+=feaafiart1ev1aaatCvAUfKttLearuWrP9MDH5MBPbIqV92AaeXatLxBI9gBamrtHrhAL1wy0L2yHvtyaeHbnfgDOvwBHrxAJfwnaebbnrfifHhDYfgasaacH8akY=wiFfYdH8Gipec8Eeeu0xXdbba9frFj0=OqFfea0dXdd9vqai=hGuQ8kuc9pgc9s8qqaq=dirpe0xb9q8qiLsFr0=vr0=vr0dc8meaabaqaciaacaGaaeqabaWaaeGaeaaakeaaimaacqWFneVtaaa@383B@^(*B*) ^using three two-layered feed-forward neural networks. In our tests the input associated with the *j*-th residue *i*_*j *_contains amino acid information, secondary structure information, and hydrophobicity interaction values described in [20]. Amino acid information is obtained from multiple sequence alignments of the protein sequence to its homologues to leverage evolutionary information. Amino acids are coded as letters out of an alphabet of 25. Beside the 20 standard amino acids, B (aspartic acid or asparagine), U (selenocysteine), X (unknown), Z (glutamic acid or glutamine) and (gap) are considered. The input presented to the networks is the frequency of each of the 24 non-gap symbols, plus the overall frequency of gaps in each column of the alignment. I.e., if *n*_*jk *_is the total number of occurrences of symbol *j *in column *k*, and *g*_*k *_the number of gaps in the same column, the *j*^*th *^input to the networks in position *k *is:

njk∑v=124nvk     (4)
 MathType@MTEF@5@5@+=feaafiart1ev1aaatCvAUfKttLearuWrP9MDH5MBPbIqV92AaeXatLxBI9gBaebbnrfifHhDYfgasaacH8akY=wiFfYdH8Gipec8Eeeu0xXdbba9frFj0=OqFfea0dXdd9vqai=hGuQ8kuc9pgc9s8qqaq=dirpe0xb9q8qiLsFr0=vr0=vr0dc8meaabaqaciaacaGaaeqabaqabeGadaaakeaadaWcaaqaaiabd6gaUnaaBaaaleaacqWGQbGAcqWGRbWAaeqaaaGcbaWaaabmaeaacqWGUbGBdaWgaaWcbaGaemODayNaem4AaSgabeaaaeaacqWG2bGDcqGH9aqpcqaIXaqmaeaacqaIYaGmcqaI0aana0GaeyyeIuoaaaGccaWLjaGaaCzcamaabmaabaGaeGinaqdacaGLOaGaayzkaaaaaa@408F@

for *j *= 1...24, while the 25^*th *^input is:

gkgk+∑v=124nvk     (5)
 MathType@MTEF@5@5@+=feaafiart1ev1aaatCvAUfKttLearuWrP9MDH5MBPbIqV92AaeXatLxBI9gBaebbnrfifHhDYfgasaacH8akY=wiFfYdH8Gipec8Eeeu0xXdbba9frFj0=OqFfea0dXdd9vqai=hGuQ8kuc9pgc9s8qqaq=dirpe0xb9q8qiLsFr0=vr0=vr0dc8meaabaqaciaacaGaaeqabaqabeGadaaakeaadaWcaaqaaiabdEgaNnaaBaaaleaacqWGRbWAaeqaaaGcbaGaem4zaC2aaSbaaSqaaiabdUgaRbqabaGccqGHRaWkdaaeWaqaaiabd6gaUnaaBaaaleaacqWG2bGDcqWGRbWAaeqaaaqaaiabdAha2jabg2da9iabigdaXaqaaiabikdaYiabisda0aqdcqGHris5aaaakiaaxMaacaWLjaWaaeWaaeaacqaI1aqnaiaawIcacaGLPaaaaaa@42F4@

This input coding scheme is richer than simple 20-letter schemes and has proven effective in [21]. The secondary structure part of the input is encoded using a three-letter scheme (helix, strand, coil). We adopt both true secondary structures, and secondary structures predicted by Porter [21]. When using predicted secondary structure, we carefully design our tests so that no sequence used for testing the PE prediction system is similar to sequences in Porter's training set. A single real-valued input is used to encode hydrophobicity interaction values. In [20] an optimised version of the scale is shown to be highly correlated to the PE. A further unit is used to encode the protein length (normalised by a factor 0.001).

Based on this encoding, a total of 30 units are used to represent each residue.

We adopt a second filtering BRNN, similarly to [21]. The network is trained to predict the PE given the first-layer PE predictions. The *i*-th input to this second network includes the first-layer predictions in position *i *augmented by first stage predictions averaged over multiple contiguous windows. I.e., if *c*_*j*1_, ... *c*_*jm *_are the outputs in position *j *of the first stage network corresponding to estimated probability of eigenvector component *j *being in class *m*, the input to the second stage network in position *j *is the array *I*_*j*_:

Ij=(cj1,…,cjm,     (6)∑h=k−p−wk−p+wch1,…,∑h=k−p−wk−p+wchm,…∑h=kp−wkp+wch1,…,∑h=kp−wkp+wchm)
 MathType@MTEF@5@5@+=feaafiart1ev1aaatCvAUfKttLearuWrP9MDH5MBPbIqV92AaeXatLxBI9gBaebbnrfifHhDYfgasaacH8akY=wiFfYdH8Gipec8Eeeu0xXdbba9frFj0=OqFfea0dXdd9vqai=hGuQ8kuc9pgc9s8qqaq=dirpe0xb9q8qiLsFr0=vr0=vr0dc8meaabaqaciaacaGaaeqabaqabeGadaaakeaafaqaceabcaaaaeaacqWGjbqsdaWgaaWcbaGaemOAaOgabeaakiabg2da9iabcIcaOiabdogaJnaaBaaaleaacqWGQbGAcqaIXaqmaeqaaOGaeiilaWIaeSOjGSKaeiilaWIaem4yam2aaSbaaSqaaiabdQgaQjabd2gaTbqabaGccqGGSaalaeaacaWLjaGaaCzcamaabmaabaGaeGOnaydacaGLOaGaayzkaaaabaWaaabCaeaacqWGJbWydaWgaaWcbaGaemiAaGMaeGymaeJaeiilaWIaeSOjGSKaeiilaWcabeaaaeaacqWGObaAcqGH9aqpcqWGRbWAdaWgaaadbaGaeyOeI0IaemiCaahabeaaliabgkHiTiabdEha3bqaaiabdUgaRnaaBaaameaacqGHsislcqWGWbaCaeqaaSGaey4kaSIaem4DaChaniabggHiLdGcdaaeWbqaaiabdogaJnaaBaaaleaacqWGObaAcqWGTbqBcqGGSaalaeqaaaqaaiabdIgaOjabg2da9iabdUgaRnaaBaaameaacqGHsislcqWGWbaCaeqaaSGaeyOeI0Iaem4DaChabaGaem4AaS2aaSbaaWqaaiabgkHiTiabdchaWbqabaWccqGHRaWkcqWG3bWDa0GaeyyeIuoaaOqaaaqaaiablAcilbqaaaqaamaaqahabaGaem4yam2aaSbaaSqaaiabdIgaOjabigdaXiabcYcaSiablAciljabcYcaSaqabaaabaGaemiAaGMaeyypa0Jaem4AaS2aaSbaaWqaaiabdchaWbqabaWccqGHsislcqWG3bWDaeaacqWGRbWAdaWgaaadbaGaemiCaahabeaaliabgUcaRiabdEha3bqdcqGHris5aOWaaabCaeaacqWGJbWydaWgaaWcbaGaemiAaGMaemyBa0gabeaakiabcMcaPaWcbaGaemiAaGMaeyypa0Jaem4AaS2aaSbaaWqaaiabdchaWbqabaWccqGHsislcqWG3bWDaeaacqWGRbWAdaWgaaadbaGaemiCaahabeaaliabgUcaRiabdEha3bqdcqGHris5aaGcbaaaaaaa@9AC2@

where *k*_*f *_= *j *+ *f*(2*w *+ 1), 2*w *+ 1 is the size of the window over which first-stage predictions are averaged and 2*p *+ 1 is the number of windows considered. In the tests we use *w *= 7 and *p *= 7. This means that 15 contiguous, non-overlapping windows of 15 residues each are considered, i.e. first-stage outputs between position *j*-112 and *j*+112, for a total of 225 contiguous residues, are taken into account to generate the input to the filtering network in position *j*. This input contains a total of 16*m *real numbers: *m *representing the *m*-class output of the first stage in position *j*; 15*m *representing the *m*-class outputs of the first-stage *averaged *over each of the 15 windows.

Five two-stage BRNN models are trained independently and ensemble averaged to build the final predictor. Differences among models are introduced by two factors: stochastic elements in the training-protocol, such as different initial weights of the networks and different shuffling of the examples; different architecture and number of free parameters of the models. Averaging the 5 models' outputs leads to classification performance improvements between 1% and 1.5% over single models. In [32] a slight improvement in secondary structure prediction accuracy was obtained by "brute ensembling" of several tens of different models trained independently. Here we adopt a less expensive technique: a copy of each of the 5 models is saved at regular intervals (100 epochs) during training. Stochastic elements in the training protocol (similar to that described in [33]) guarantee that differences during training are non-trivial. When an ensemble of 9 such copies for all the 5 models is used (45 models in total) we obtain a further slight improvement over the ensemble of 5 models.

### Predictive architecture for contact maps

We build a system for the prediction of contact maps based on 2D-RNN, described in [4] and [13]. This is a family of adaptive models for mapping two-dimensional matrices of variable size into matrices of the same size. 2D-RNN-based models were among the most successful contact map predictors at the CASP5 competition [27].

As in the PE prediction case, we use 2D-RNNs with *shortcut connections*, i.e. where lateral memory connections span *N*-residue intervals, where *N *> 1. If *o*_*j*,*k *_is the entry in the *j*-th row and *k*-th column of the output matrix (in our case, it will represent the estimated probability of residues *j *and *k *being in contact), and *i*_*j*,*k *_is the input in the same position, the input-output mapping is modelled as:

oj,k=N(O)(ij,k,hj,k(1),hj,k(2),hj,k(3),hj,k(4))hj,k(1)=N(1)(ij,k,hj−1,k(1),..,hj−S,k(1),hj,k−1(1),..,hj,k−S(1))hj,k(2)=N(2)(ij,k,hj+1,k(2),..,hj+S,k(2),hj,k−1(2),..,hj,k−S(2))hj,k(3)=N(3)(ij,k,hj+1,k(3),..,hj+S,k(3),hj,k+1(3),..,hj,k+S(3))hj,k(4)=N(4)(ij,k,hj−1,k(4),..,hj−S,k(4),hj,k+1(4),..,hj,k+S(4))j,k=1,…,N
 MathType@MTEF@5@5@+=feaafiart1ev1aaatCvAUfKttLearuWrP9MDH5MBPbIqV92AaeXatLxBI9gBamrtHrhAL1wy0L2yHvtyaeHbnfgDOvwBHrxAJfwnaebbnrfifHhDYfgasaacH8akY=wiFfYdH8Gipec8Eeeu0xXdbba9frFj0=OqFfea0dXdd9vqai=hGuQ8kuc9pgc9s8qqaq=dirpe0xb9q8qiLsFr0=vr0=vr0dc8meaabaqaciaacaGaaeqabaWaaeGaeaaakqGabeqaaG3abaGaem4Ba82aaSbaaSqaaiabdQgaQjabcYcaSiabdUgaRbqabaGccqGH9aqpimaacqWFneVtdaahaaWcbeqaamaabmaabaGaem4ta8eacaGLOaGaayzkaaaaaOWaaeWaaeaacqWGPbqAdaWgaaWcbaGaemOAaOMaeiilaWIaem4AaSgabeaakiabcYcaSiabdIgaOnaaDaaaleaacqWGQbGAcqGGSaalcqWGRbWAaeaadaqadaqaaiabigdaXaGaayjkaiaawMcaaaaakiabcYcaSiabdIgaOnaaDaaaleaacqWGQbGAcqGGSaalcqWGRbWAaeaadaqadaqaaiabikdaYaGaayjkaiaawMcaaaaakiabcYcaSiabdIgaOnaaDaaaleaacqWGQbGAcqGGSaalcqWGRbWAaeaadaqadaqaaiabiodaZaGaayjkaiaawMcaaaaakiabcYcaSiabdIgaOnaaDaaaleaacqWGQbGAcqGGSaalcqWGRbWAaeaadaqadaqaaiabisda0aGaayjkaiaawMcaaaaaaOGaayjkaiaawMcaaaqaaiabdIgaOnaaDaaaleaacqWGQbGAcqGGSaalcqWGRbWAaeaadaqadaqaaiabigdaXaGaayjkaiaawMcaaaaakiabg2da9iab=1q8onaaCaaaleqabaWaaeWaaeaacqaIXaqmaiaawIcacaGLPaaaaaGcdaqadaqaaiabdMgaPnaaBaaaleaacqWGQbGAcqGGSaalcqWGRbWAaeqaaOGaeiilaWIaemiAaG2aa0baaSqaaiabdQgaQjabgkHiTiabigdaXiabcYcaSiabdUgaRbqaamaabmaabaGaeGymaedacaGLOaGaayzkaaaaaOGaeiilaWIaeiOla4IaeiOla4IaeiilaWIaemiAaG2aa0baaSqaaiabdQgaQjabgkHiTiabdofatjabcYcaSiabdUgaRbqaamaabmaabaGaeGymaedacaGLOaGaayzkaaaaaOGaeiilaWIaemiAaG2aa0baaSqaaiabdQgaQjabcYcaSiabdUgaRjabgkHiTiabigdaXaqaamaabmaabaGaeGymaedacaGLOaGaayzkaaaaaOGaeiilaWIaeiOla4IaeiOla4IaeiilaWIaemiAaG2aa0baaSqaaiabdQgaQjabcYcaSiabdUgaRjabgkHiTiabdofatbqaamaabmaabaGaeGymaedacaGLOaGaayzkaaaaaaGccaGLOaGaayzkaaaabaGaemiAaG2aa0baaSqaaiabdQgaQjabcYcaSiabdUgaRbqaamaabmaabaGaeGOmaidacaGLOaGaayzkaaaaaOGaeyypa0Jae8xdX70aaWbaaSqabeaadaqadaqaaiabikdaYaGaayjkaiaawMcaaaaakmaabmaabaGaemyAaK2aaSbaaSqaaiabdQgaQjabcYcaSiabdUgaRbqabaGccqGGSaalcqWGObaAdaqhaaWcbaGaemOAaOMaey4kaSIaeGymaeJaeiilaWIaem4AaSgabaWaaeWaaeaacqaIYaGmaiaawIcacaGLPaaaaaGccqGGSaalcqGGUaGlcqGGUaGlcqGGSaalcqWGObaAdaqhaaWcbaGaemOAaOMaey4kaSIaem4uamLaeiilaWIaem4AaSgabaWaaeWaaeaacqaIYaGmaiaawIcacaGLPaaaaaGccqGGSaalcqWGObaAdaqhaaWcbaGaemOAaOMaeiilaWIaem4AaSMaeyOeI0IaeGymaedabaWaaeWaaeaacqaIYaGmaiaawIcacaGLPaaaaaGccqGGSaalcqGGUaGlcqGGUaGlcqGGSaalcqWGObaAdaqhaaWcbaGaemOAaOMaeiilaWIaem4AaSMaeyOeI0Iaem4uamfabaWaaeWaaeaacqaIYaGmaiaawIcacaGLPaaaaaaakiaawIcacaGLPaaaaeaacqWGObaAdaqhaaWcbaGaemOAaOMaeiilaWIaem4AaSgabaWaaeWaaeaacqaIZaWmaiaawIcacaGLPaaaaaGccqGH9aqpcqWFneVtdaahaaWcbeqaamaabmaabaGaeG4mamdacaGLOaGaayzkaaaaaOWaaeWaaeaacqWGPbqAdaWgaaWcbaGaemOAaOMaeiilaWIaem4AaSgabeaakiabcYcaSiabdIgaOnaaDaaaleaacqWGQbGAcqGHRaWkcqaIXaqmcqGGSaalcqWGRbWAaeaadaqadaqaaiabiodaZaGaayjkaiaawMcaaaaakiabcYcaSiabc6caUiabc6caUiabcYcaSiabdIgaOnaaDaaaleaacqWGQbGAcqGHRaWkcqWGtbWucqGGSaalcqWGRbWAaeaadaqadaqaaiabiodaZaGaayjkaiaawMcaaaaakiabcYcaSiabdIgaOnaaDaaaleaacqWGQbGAcqGGSaalcqWGRbWAcqGHRaWkcqaIXaqmaeaadaqadaqaaiabiodaZaGaayjkaiaawMcaaaaakiabcYcaSiabc6caUiabc6caUiabcYcaSiabdIgaOnaaDaaaleaacqWGQbGAcqGGSaalcqWGRbWAcqGHRaWkcqWGtbWuaeaadaqadaqaaiabiodaZaGaayjkaiaawMcaaaaaaOGaayjkaiaawMcaaaqaaiabdIgaOnaaDaaaleaacqWGQbGAcqGGSaalcqWGRbWAaeaadaqadaqaaiabisda0aGaayjkaiaawMcaaaaakiabg2da9iab=1q8onaaCaaaleqabaWaaeWaaeaacqaI0aanaiaawIcacaGLPaaaaaGcdaqadaqaaiabdMgaPnaaBaaaleaacqWGQbGAcqGGSaalcqWGRbWAaeqaaOGaeiilaWIaemiAaG2aa0baaSqaaiabdQgaQjabgkHiTiabigdaXiabcYcaSiabdUgaRbqaamaabmaabaGaeGinaqdacaGLOaGaayzkaaaaaOGaeiilaWIaeiOla4IaeiOla4IaeiilaWIaemiAaG2aa0baaSqaaiabdQgaQjabgkHiTiabdofatjabcYcaSiabdUgaRbqaamaabmaabaGaeGinaqdacaGLOaGaayzkaaaaaOGaeiilaWIaemiAaG2aa0baaSqaaiabdQgaQjabcYcaSiabdUgaRjabgUcaRiabigdaXaqaamaabmaabaGaeGinaqdacaGLOaGaayzkaaaaaOGaeiilaWIaeiOla4IaeiOla4IaeiilaWIaemiAaG2aa0baaSqaaiabdQgaQjabcYcaSiabdUgaRjabgUcaRiabdofatbqaamaabmaabaGaeGinaqdacaGLOaGaayzkaaaaaaGccaGLOaGaayzkaaaabaGaaCzcaiabdQgaQjabcYcaSiabdUgaRjabg2da9iabigdaXiabcYcaSiablAciljabcYcaSiabd6eaobaaaa@81CB@

where hj,k(n)
 MathType@MTEF@5@5@+=feaafiart1ev1aaatCvAUfKttLearuWrP9MDH5MBPbIqV92AaeXatLxBI9gBaebbnrfifHhDYfgasaacH8akY=wiFfYdH8Gipec8Eeeu0xXdbba9frFj0=OqFfea0dXdd9vqai=hGuQ8kuc9pgc9s8qqaq=dirpe0xb9q8qiLsFr0=vr0=vr0dc8meaabaqaciaacaGaaeqabaqabeGadaaakeaacqWGObaAdaqhaaWcbaGaemOAaOMaeiilaWIaem4AaSgabaWaaeWaaeaacqWGUbGBaiaawIcacaGLPaaaaaaaaa@34BC@ for *n *= 1, ..., 4 are planes of hidden vectors transmitting contextual information from each corner of the matrix to the opposite corner. We parametrise the output update, and the four lateral update functions (respectively N
 MathType@MTEF@5@5@+=feaafiart1ev1aaatCvAUfKttLearuWrP9MDH5MBPbIqV92AaeXatLxBI9gBamrtHrhAL1wy0L2yHvtyaeHbnfgDOvwBHrxAJfwnaebbnrfifHhDYfgasaacH8akY=wiFfYdH8Gipec8Eeeu0xXdbba9frFj0=OqFfea0dXdd9vqai=hGuQ8kuc9pgc9s8qqaq=dirpe0xb9q8qiLsFr0=vr0=vr0dc8meaabaqaciaacaGaaeqabaWaaeGaeaaakeaaimaacqWFneVtaaa@383B@^(*O*) ^and N
 MathType@MTEF@5@5@+=feaafiart1ev1aaatCvAUfKttLearuWrP9MDH5MBPbIqV92AaeXatLxBI9gBamrtHrhAL1wy0L2yHvtyaeHbnfgDOvwBHrxAJfwnaebbnrfifHhDYfgasaacH8akY=wiFfYdH8Gipec8Eeeu0xXdbba9frFj0=OqFfea0dXdd9vqai=hGuQ8kuc9pgc9s8qqaq=dirpe0xb9q8qiLsFr0=vr0=vr0dc8meaabaqaciaacaGaaeqabaWaaeGaeaaakeaaimaacqWFneVtaaa@383B@^(*n*) ^for *n *= 1, ..., 4) using five two-layered feed-forward neural networks, as in [13].

In our tests the input *i*_*j*,*k *_contains amino acid information, secondary structure and solvent accessibility information, and PE information for the amino acids in positions *j *and *k *in the sequence. Amino acid information is again obtained from multiple sequence alignments.

## Implementation

### Data set generation and input data

The data set used in the present simulations is extracted from the December 2003 25% pdb_select list [34]. We use the DSSP program [35] (CMBI version) to assign relevant structural features (true secondary structure, used in preliminary tests, and *C*_*α *_coordinates – the latter also directly available from the PDB files) and remove sequences for which DSSP does not produce an output due, for instance, to missing entries or format errors. After processing by DSSP, the set contains 2171 protein and 344,653 amino acids. Multiple sequence alignments for the 2171 proteins are extracted from the NR database as available on March 3 2004 containing over 1.4 million sequences. The database is first redundancy reduced at a 98% threshold, leading to a final 1.05 million sequences. The alignments are generated by three runs of PSI-BLAST [36] with parameters *b *= 3000, *e *= 10^-3 ^and *h *= l0^-10^.

### Experimental Protocol

For our experiments we split the data into a training set containing 1736 sequences and a test set of 435 (1/5 of the total). The test set sequences are selected in an interleaved fashion (i.e. every fifth sequence is picked) from the whole set sorted alphabetically by PDB code – this is meant to avoid biases.

For the prediction of contact maps the sets are further selected, by excluding proteins longer than 200 residues. This leaves 1275 proteins in the training set and 327 proteins in the test set.

**Figure 1:**
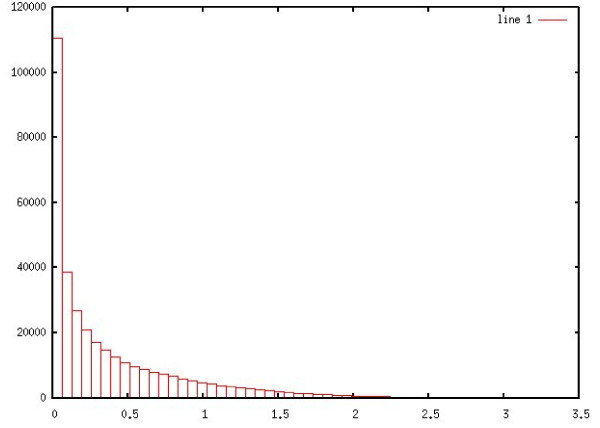
**Distribution of **λ¯x¯
 MathType@MTEF@5@5@+=feaafiart1ev1aaatCvAUfKttLearuWrP9MDH5MBPbIqV92AaeXatLxBI9gBaebbnrfifHhDYfgasaacH8akY=wiFfYdH8Gipec8Eeeu0xXdbba9frFj0=OqFfea0dXdd9vqai=hGuQ8kuc9pgc9s8qqaq=dirpe0xb9q8qiLsFr0=vr0=vr0dc8meaabaqaciaacaGaaeqabaqabeGadaaakeaacuaH7oaBgaqeaiqbdIha4zaaraaaaa@3009@_*i *_**values in the training set**. See text for details.

**Figure 2:**
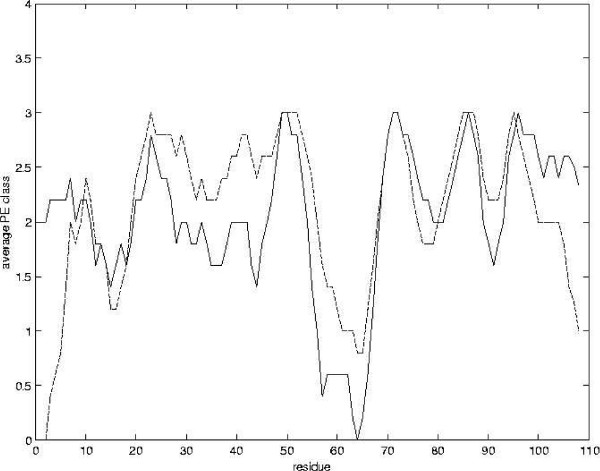
**4-class principal eigenvector prediction for protein 1A2P (108 amino acids)**. Solid line: exact eigenvector class. Dashed line: predicted eigenvector class. The class value is averaged over a moving window of 5 residues.

**Figure 3:**
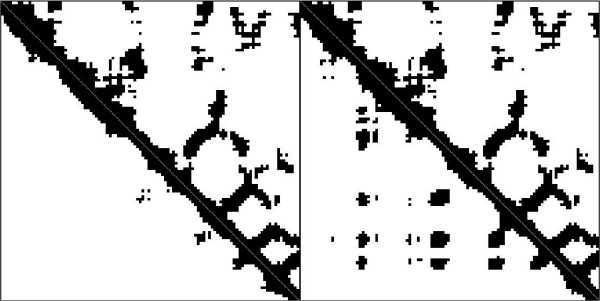
**Examples of contact map predictions at 12 Å for protein 1A2P (108 amino acids)**. Exact map in the top-right half, predicted map in the bottom-left half. Prediction by MA_SS_ACC on the left, MA_SS_ACC_PE on the right (see text for details).

To define the PE, two segments are considered in contact if the distance between their *C*_*α *_is smaller than 8 Å. As in [18], the main diagonal of the contact map (|*i *- *j*| < 3) is removed before computing the principal eigenvectors. The distribution of the components of the magnified eigenvectors λ¯x¯
 MathType@MTEF@5@5@+=feaafiart1ev1aaatCvAUfKttLearuWrP9MDH5MBPbIqV92AaeXatLxBI9gBaebbnrfifHhDYfgasaacH8akY=wiFfYdH8Gipec8Eeeu0xXdbba9frFj0=OqFfea0dXdd9vqai=hGuQ8kuc9pgc9s8qqaq=dirpe0xb9q8qiLsFr0=vr0=vr0dc8meaabaqaciaacaGaaeqabaqabeGadaaakeaacuaH7oaBgaqeaiqbdIha4zaaraaaaa@3009@_*i *_is shown in Figure 1. We attempt three distinct classification schemes: in 2, 3 and 4 classes. The class thresholds are assigned so that the examples in the training set are equally split among them: 0.179195 for the 2-class case (classes of roughly 138, 000 examples each), 0.0688848 and 0.38165 for three classes (≈ 92,000 examples each) and 0.0358246, 0.179195 and 0.541445 for 4 classes (≈ 69, 000 examples each). Both BRNNs are trained by minimising the cross-entropy error between the output and target probability distributions, using gradient descent with no momentum term or weight decay. The gradient is computed using the Back-propagation through structure (BPTS) algorithm (for which, see e.g. [37]). We use a hybrid between online and batch training, with 580 batch blocks (roughly 3 proteins each) per training set. Thus, the weights are updated 580 times per epoch. The training set is also shuffled at each epoch, so that the error does not decrease monotonically. When the error does not decrease for 50 consecutive epochs, the learning rate is divided by 2. Training stops after 1000 epochs. First-layer and filtering BRNNs are trained simultaneously, but supervised independently.

The DAG-RNNs composing the contact map predictors are trained by minimising the cross-entropy error between the output and target probability distributions. This is obtained by a modified form of gradient descent where the update for a network weight is piecewise linear in three different ranges, to avoid initial plateau problems (for a more detailed description see [13]). The gradient is computed using the BPTS algorithm [37]. Similarly to the PE case, we use a hybrid between online and batch training, with 600 batch blocks (roughly 2 proteins each) per training set. Training runs for 120 epochs, with a fixed learning rate, and the training set is shuffled after each epoch. Three networks are saved, at epoch 110, 115 and 120, and ensemble averaged to produce the final predictor.

Training the 8 predictors described in the paper took approximately a month on a cluster of 8 2.8 GHz CPUs.

## Authors' contributions

AV contributed the initial idea of predicting the principal eigenvector, and analysed the PE results. IW designed, trained and tested the predictor of contact maps. GP designed, trained and tested the PE predictive architecture, and suggested the two-stage approach for the prediction of contact maps. The manuscript was written by AV and GP, and read and approved by all authors.

## Figures and Tables

**Table 1:** PE prediction: two-class problem. Accuracy estimates with 95% confidence intervals and SOV. A * in the first three columns of a row indicates whether the results are obtained augmenting the network input with secondary structure predicted by Porter (P), hydrophobicity profile using the interactivity scale of [20] (H) and using second stage filtering network (F).

P	H	F	*Q*_2_	*Q*_0_	*Q*_1_	SOV
-	-	-	72.0 ± .6	73.1 ± .6	70.8 ± .6	44.4
-	-	*	72.1 ± .6	73.4 ± .6	70.7 ± .6	46.0
*	-	-	72.3 ± .6	73.1 ± .6	71.4 ± .6	47.6
*	-	*	72.3 ± .6	72.8 ± .6	71.9 ± .6	49.6
*	*	-	72.5 ± .6	74.0 ± .6	71.0 ± .6	47.2
*	*	*	72.6 ± .6	73.8 ± .6	71.2 ± .6	49.8
baseline			56.8 ± .5	58.4 ± .5	55.1 ± .5	-

**Table 2:** PE prediction: three-class problem. Accuracy estimates with 95% confidence intervals and SOV. A * in the first three columns of a row indicates whether the results are obtained augmenting the network input with secondary structure predicted by Porter (P), hydrophobicity profile using the interactivity scale of [20] (H) and using second stage filtering network (F).

P	H	F	*Q*_3_	*Q*_0_	*Q*_1_	*Q*_2_	SOV
-	-	-	55.8 ± .5	63.7 ± .5	35.5 ± .4	67.6 ± .6	38.3
-	-	*	56.2 ± .5	61.6 ± .6	40.6 ± .5	65.8 ± .6	40.3
*	-	-	56.3 ± .6	65.8 ± .6	36.9 ± .5	65.5 ± .6	42.1
*	-	*	56.6 ± .6	64.1 ± .6	41.1 ± .4	63.9 ± .6	43.6
*	*	-	56.4 ± .5	65.2 ± .6	36.8 ± .4	66.4 ± .6	42.6
*	*	*	56.7 ± .5	63.3 ± .6	41.4 ± .4	64.6 ± .6	44.0
baseline			39.8 ± .4	50.6 ± .5	8.5 ± .2	59.3 ± .6	-

**Table 3:** PE prediction: four-class problem. Accuracy estimates with 95% confidence intervals and SOV. A * in the first three columns of a row indicates whether the results are obtained augmenting the network input with secondary structure predicted by Porter (P), hydrophobicity profile using the interactivity scale of [20] (H) and using second stage filtering network (F).

P	H	F	*Q*_4_	*Q*_0_	*Q*_1_	*Q*_2_	*Q*_3_	SOV
-	-	-	45.6 ± .5	59.9 ± .6	29.4 ± .4	26.8 ± .4	65.0 ± .6	33.2
-	-	*	46.0 ± .5	58.5 ± .5	30.6 ± .4	30.5 ± .4	63.3 ± .6	34.6
*	-	-	46.2 ± .5	62.1 ± .6	30.5 ± .4	27.3 ± .4	63.1 ± .6	37.3
*	-	*	46.5 ± .5	60.7 ± .5	32.3 ± .4	29.6 ± .4	61.8 ± .5	37.4
*	*	-	45.9 ± .5	61.5 ± .5	30.0 ± .4	27.3 ± .3	63.3 ± .6	37.1
*	*	*	46.5 ± .5	60.0 ± .5	32.2 ± .4	30.4 ± .4	61.9 ± .6	37.8
baseline			30.7 ± .4	48.1 ± .5	5.4 ± .2	8.5 ± .2	59.0 ± .6	-

**Table 4:** Performance results for contact map prediction. Contact threshold: 8 Å. Accuracy, coverage and F1 (as %) for 8 Å contact map predictor for distance separations greater than 5, 11 and 23 amino acids.

	|*i *- *j*| ≥ 6				|*i *- *j*| ≥ 12				|*i *- *j*| ≥ 24			
	
	P	R	F_1_	P_*nc*_	P	R	F_1_	P_*nc*_	P	R	F_1_	P_*nc*_
MA	0	0	0	97.2	0	0	0	97.6	0	0	0	97.9
MA_PE	39.4	12.2	18.6	97.5	36.2	8.4	13.5	97.7	27.8	2.0	3.7	97.9
MA_SS_ACC	50.5	7.4	12.9	97.4	48.8	4.0	7.4	97.6	25.7	.2	.3	97.9
MA_SS_ACC_PE	43.3	11.3	17.9	97.5	38.9	7.2	12.1	97.7	25.5	2.2	4.1	97.9

**Table 5:** Performance results for contact map prediction. Contact threshold: 8 Å. Accuracy and coverage (as %) for 8 Å contact map predictor for distance separations greater than 5, 11 and 23 amino acids, when the top *N*/5 and top *N*/2 contacts are considered (where *N *is the length of the protein).

	|*i *- *j*| ≥ 6				|*i *- *j*| ≥ 12				|*i *- *j*| ≥ 24			
	
	*N*/5		*N*/2		*N*/5		*N*/2		*N*/5		*N*/2	
	
	P	R	P	R	P	R	P	R	P	R	P	R
MA	30.8	3.9	26.2	8.5	23.9	3.8	19.2	7.8	14.1	3.3	11.1	6.6
MA_PE	43.0	5.5	34.6	11.2	34.2	5.5	26.6	10.8	19.6	4.6	15.0	8.9
MA_SS_ACC	44.4	5.7	36.0	11.6	34.2	5.5	26.6	10.8	17.8	4.2	14.7	8.7
MA_SS_ACC_PE	46.4	5.9	36.6	11.8	35.4	5.7	27.0	11.0	19.8	4.6	15.7	9.3

**Table 6:** Performance results for contact map prediction. Contact threshold: 12 Å. Accuracy, coverage and F1 (as %) for 12 Å contact map predictor for distance separations greater than 5, 11 and 23 amino acids.

	|*i *- *j*| ≥ 6				|*i *- *j*| ≥ 12				|*i *- *j*| ≥ 24			
	
	P	R	F_1_	P_*nc*_	P	R	F_1_	P_*nc*_	P	R	F_1_	P_*nc*_
MA	60.4	10.6	18.1	87.2	55.8	0.1	0.1	87.8	38.9	0.03	0.06	88.8
MA_PE	49.5	24.5	32.8	88.6	39.4	16.8	23.6	89.3	34.5	13.6	19.5	89.9
MA_SS_ACC	61.6	19.6	29.7	88.2	48.9	7.5	13.1	88.5	40.2	2.8	5.3	89.0
MA_SS_ACC_PE	54.2	23.5	32.8	88.6	42.2	14.6	21.7	89.2	36.7	10.9	16.8	89.7

**Table 7:** Performance results for contact map prediction. Contact threshold: 12 Å. Accuracy and coverage (as %) for 12 Å contact map predictor for distance separations greater than 5, 11 and 23 amino acids, when the top *N*/5 and top *N*/2 contacts are considered (where *N *is the length of the protein).

	|*i *- *j*| ≥ 6				|*i *- *j*| ≥ 12				|*i *- *j*| ≥ 24			
	
	*N*/5		*N*/2		*N*/5		*N*/2		*N*/5		*N*/2	
	
	P	R	P	R	P	R	P	R	P	R	P	R
MA	79.6	2.0	71.9	4.6	50.1	1.6	46.2	3.8	43.3	1.9	38.1	4.2
MA_PE	87.5	2.2	81.6	5.2	59.8	1.9	54.1	4.4	47.9	2.1	42.4	4.7
MA_SS_ACC	89.7	2.3	85.3	5.5	61.3	2.0	54.9	4.5	43.7	1.9	39.4	4.4
MA_SS_ACC_PE	89.9	2.3	85.5	5.5	62.5	2.0	55.6	4.6	49.9	2.2	43.8	4.9
